# The Azurin Coding Gene: Origin and Phylogenetic Distribution

**DOI:** 10.3390/microorganisms10010009

**Published:** 2021-12-22

**Authors:** Leandro Gammuto, Carolina Chiellini, Marta Iozzo, Renato Fani, Giulio Petroni

**Affiliations:** 1 Department of Biology, University of Pisa, 56126 Pisa, Italy; leandro.g@hotmail.it; 2 National Research Council, Institute of Agricultural Biology and Biotechnology, Via Moruzzi 1, 56124 Pisa, Italy; chiellini.carolina@gmail.com; 3 Department of Experimental and Clinical Biomedical Sciences, University of Florence, Viale Morgagni 50, 50134 Florence, Italy; marta.iozzo@student.unisi.it; 4 Laboratory of Microbial and Molecular Evolution, Department of Biology, University of Florence, Via Madonna del Piano 6, 50019 Sesto Fiorentino, Italy

**Keywords:** azurin, phylogeny, bacteria, *Proteobacteria*, *Bacteroidetes*, *Verrucomicrobia*, genomics, p28

## Abstract

Azurin is a bacterial-derived cupredoxin, which is mainly involved in electron transport reactions. Interest in azurin protein has risen in recent years due to its anticancer activity and its possible applications in anticancer therapies. Nevertheless, the attention of the scientific community only focused on the azurin protein found in *Pseudomonas aeruginosa* (*Proteobacteria*, *Gammaproteobacteria*). In this work, we performed the first comprehensive screening of all the bacterial genomes available in online repositories to assess azurin distribution in the three domains of life. The Azurin coding gene was not detected in the domains Archaea and Eucarya, whereas it was detected in phyla other than *Proteobacteria*, such as *Bacteroidetes*, *Verrucomicrobia* and *Chloroflexi,* and a phylogenetic analysis of the retrieved sequences was performed. Observed patchy distribution and phylogenetic data suggest that once it appeared in the bacterial domain, the azurin coding gene was lost in several bacterial phyla and/or anciently horizontally transferred between different phyla, even though a vertical inheritance appeared to be the major force driving the transmission of this gene. Interestingly, a shared conserved domain has been found among azurin members of all the investigated phyla. This domain is already known in *P. aeruginosa* as p28 domain and its importance for azurin anticancer activity has been widely explored. These findings may open a new and intriguing perspective in deciphering the azurin anticancer mechanisms and to develop new tools for treating cancer diseases.

## 1. Introduction

The ability of several bacterial secondary metabolites to affect tumor cell survival and/or their growth and replication ratio, has gained the attention of scientists for many years [1,2,3,4,5]. Members of the metalloprotein family named cupredoxins are among the wide range of molecules, either of proteinaceous or non-proteinaceous nature, representing putative candidates for such purpose, and identified during this long-lasting research. Cupredoxins, also known as blue copper proteins, are small soluble proteins (around 14 kDa) involved in electron transport reactions in bacteria and plants [6,7], which bind in their active site a type-1 copper. This metal ligand gives them the blue colour, which is responsible for their name. The cupredoxin protein family includes various subfamilies, such as auracyanins, amicyanins, azurins, rusticyanins, pseudoazurins, and halocyanins [8,9]. Even if members of these subfamilies show a rather low degree of sequence identity (less than 20%), they all share significant structural similarities. In fact, they all possess eight parallel and antiparallel strands, composing a b-barrel formation or a b-sandwich fold, and a highly conserved structure regarding their active site [6,7]. In all the cupredoxins, the copper metal binds to the protein in correspondence with the b-barrel, contacting 1 cysteine, 2 histidines, and 1 methionine residue in a tetrahedral conformation [10]. Among cupredoxins, one of the most extensively studied proteins is azurin, a small water-soluble periplasmic protein composed of four identical monomers each of about 150 amino acids. This protein is mainly involved in the bacterial denitrification processes, acting as electron donors for the nitrite reductase [11]. Azurin was discovered in *Pseudomonas aeruginosa* in 1956 [12] and first purified in 1958 [13]. Other than acting in the denitrification process, azurin had been found to possess anticancer activity and many studies have highlighted that azurin preferentially enters cancer cells rather than healthy ones [14,15]. This capability is linked to the so-called p28 domain, which roughly corresponds to the peptide Leu50-Asp77 on *Pseudomonas aeruginosa* azurin that forms the extended amphipathic alpha-helical region linking the beta strands and represents the transport domain of the protein. The p28 domain interacts with cholesterol microdomains (lipid rafts), which are overexpressed on the cell membrane of cancer cells [16], allowing the preferential entry of azurin inside cancer cells via endocytic pathway [17,18]. Once inside the cancer cell, Azurin exploits its anticancer activity complexing with p53 and preventing its degradation [19]. The complex formed by azurin and p53 is imported inside the nucleus, where p53 can upregulate the expression of pro-apoptotic genes such as *Noxa* and *Baxa* [19], leading to the activation of the apoptotic machinery via releasing cytochrome c in the cytosol [20,21]. Therefore azurin, as well as the p28 domain, gained great attention by the researchers due to its anticancer activity and possible use in cancer disease treatments. Nevertheless, almost all the studies to date, only focus on the azurin protein produced by *P. aeruginosa*. Indeed *P. aeruginosa* azurin had been deeply studied from a functional and structural viewpoint [3,22,23,24,25]. At the same time, very little is known about its presence in other bacterial genera, or about its evolutionary history and its role in other organisms rather than *P. aeruginosa*. Up to now, azurin presence had been reported as being limited to members of phylum *Proteobacteria*, such as representatives of genera *Bordetella* [26], *Pseudomonas* and *Alcaligens* [27], but to the best of our knowledge, no comprehensive screening for the presence of azurin in bacterial genomes has yet been undertaken. In this work, we screened, for the first time, all the available bacterial, archaeal, and eukaryotic genomes that are present on the NCBI online database, expanding the knowledge about the distribution of azurin, and azurin-like proteins, in bacteria, and exploring, at the same time, their evolutionary history. Given the interest gained by azurin in recent decades, especially in clinical studies, we think that our work might be useful for applied purposes in the therapeutic field, setting the base for further investigation focused on the azurin retrieved in different taxonomic groups other than *Pseudomonas aeruginosa*. Accordingly, the results herein presented will open the way for further evaluation in pre-clinical and clinical studies of similar anticancer activity of azurin from different bacterial groups, as well as the putative investigation on novel and interesting properties of these secondary metabolites.

## 2. Materials and Methods

The protein coding sequences of all the available bacterial genomes were downloaded from NCBI online database, using the “RDP—Taxonomy Browser” [28]. According to the annotation on NCBI Taxonomy, the downloaded sequences were divided according to their phylum rank. Bacterial genomes were downloaded regardless of their assembly status (e.g., chromosome, complete, scaffold, contigs etc), or their source (e.g., shotgun sequencing, derived from metagenomic analyzes, etc.). To detect the presence of azurin-like genes, a local protein database was made for each phylum, using protein sequences, and a blastp analysis was performed using *P. aeruginosa* PA01 azurin gene (ACC P00282) as query. E-value threshold for significance was set to 1 e-05. Then a manual refining of the hits list was made, excluding all hits longer than 200 amino acids (aa) and shorter than 100 aa. Most divergent sequences were manually checked to avoid false positives (e.g., auracyanin and/or cytochrome-c precursor). For a list of all the retrieved hits and related accession numbers see Supplementary Table S1. An identical approach has been used to also screen eukaryotic and archaeal genomes (19424 and 5291, respectively) for the presence of azurin genes. All the available eukaryotic and archaeal genomes were downloaded using the “RDP—Taxonomy Browser”, and a blastp analyzes was performed using *P. aeruginosa* PA01 azurin amino acid sequence (ACC P00282) as a query. To evaluate the number of positive genomes and the number of azurin hits on each genome, the related BioSample accession number for each hit (when available) was downloaded from the NCBI BioSample database (http://www.ncbi.nlm.nih.gov/biosample/, accessed on 15 November 2021). Each different BioSample was considered as a different genome, and hits related to the same BioSample were considered as belonging to the same genome.

Mafft software version 7.48 [29] was used to create a multi-alignment of the retrieved amino acidic sequences using a hierarchical approach. Sequences belonging to each phylum were aligned separately and, subsequently, the obtained multi-alignments were aligned to each other limiting the number of changes among sequences belonging to the same phylum. ARB software version 5.5 [30] was used to visualize and manage the sequence alignment and to perform a manual refining. Sequences were then trimmed to the shortest sequence, and a positional filter to exclude sites with a degree of identity lower than 5%, non-considering gaps as an additional character for the purpose of minimal identity calculation, was applied. The resulting matrix was composed of 185 sites. Fasttree software version 2.1 [31] was used to construct the Maximum-Likelihood tree, using software default parameters.

To investigate whether azurin-like genes share a common operon structure across the various phyla, for *Bacteroidetes*, *PVC*, *Proteobacteria* and *Acidobacteria*, 10 randomly selected, complete genomes (or all the available if less than 10 identified genomes) were screened for each retrieved group of organisms about the presence of an azurin -related operon and analyzed with Operon-mapper web-tool [32]. The final selection is composed of 10 complete genomes belonging to phylum *Proteobacteria*, 10 complete genomes belonging to phylum *Bacteroidetes*, 8 complete genomes belonging to *PVC* superphylum and 2 complete genomes belonging to *Acidobacteria* (a complete list of analyzed genomes can be found in Supplementary Materials).

Starting from the same sequence selection used for the phylogenetic analyzes, local implementation of MEME-suite [33] was used to detect the presence of any conserved motifs across the investigated sequences. To investigate the presence of analogous p28 domains, FITO software [34] was used, using the p28 fragment found in *P. aeruginosa* azurin gene as a reference. GLAM2 [35] was used on the so-retrieved sequences for the p28 fragments to assess the level of conservation inside each phylum and among phyla.

## 3. Results

### 3.1. Distribution of Azurin Coding Genes

The phylogenetic distribution of azurin was checked across the three domains of life, as described in Material and Methods using the *P. aeruginosa* PA01 azurin amino acid sequence as query. Data obtained are shown in Table 1, wherein analysis revealed that the presence of azurin was limited to some bacterial phyla. Indeed, the screening for azurin presence in eukaryotic and archaeal genomes resulted in just 4 and 3 positive hits, respectively. A closer inspection of the hits revealed that not only the genomes harbouring those genes were derived from metagenomic project and were very fragmented, but also that the assembled sequences harbouring azurin genes were very short, with very low coverage and harbouring other bacterial genes. On this basis, it is quite possible that all the few archaeal and eukaryotic hits might belong to bacterial contaminants, and no azurin genes were therefore present on archaeal and eukaryotic genomes.

Concerning the domain Bacteria, azurin genes were detected in 5 main phylum-like clades, i.e., *Proteobacteria, Bacteroidetes*, and other members of the FCB group, *PVC* superphylum, *Terrabacteria* group (i.e., *Chloroflexi* and *Actinobacteria*) and *Acidobacteria*. The highest number of hits were found in genomes belonging to *Proteobacteria* (Table 1), mainly present in *Betaproteobacteria* and *Gammaproteobacteria* (Table 1), even though hits for azurin-encoding genes were also found in few genome sequences attributed to *Deltaproteobacteria* and *Alphaproteobacteria* (Table 1).

Taking into consideration also, the atypical phylogenetic clustering of many Azurin *Alphaproteobacteria* sequences (see later figure), contigs containing these gene were further analyzed to confirm their correct taxonomic attribution, i.e., we considered “verified hits” only those retrieved in large contigs in which the phylogenetic signal from other genes allowed us to attribute the contig to *Alphaproteobacteria* or when no clear taxonomical attribution could be made. Only a minority of these sequences (Supplementary Table S2) fulfilled these criteria, supporting the idea that the presence of azurin in *Alphaproteobacteria* should be considered with extreme caution.

The second most represented group is the FCB one (Table 1). Almost all the identified hits were found in sequences belonging to *Bacteroidetes*, with the exception of four hits associated with the phylum *Gemmatimonadetes* (Table 1). Inside the *PVC* superphylum almost all the hits were found in sequences belonging to members of the phylum *Verrucomicrobia*, with the only exception of one hit on a draft genome belonging to *Planctomycetes* (Table 1D). Lastly, inside the group *Terrabacteria,* two phyla showed hits for azurin, i.e., *Chloroflexi* and *Actinobacteria*, with the first by far the most represented (Table 1E.). Interestingly, no azurin sequence was found in *Cyanobacteria*, *Firmicutes*, *Tenericutes*, and others, despite of the large number of genomes scanned (1794, 145,162, 1173, and 741, respectively). In all cases in which we observed a limited number of azurin genes in a specific taxonomic group, further analysis similar to those performed for *Alphaproteobacteria* were performed (i.e., on *Gemmatimonadetes*, *Planctomycetes* and *Actinobacteria*, see Supplementary Table S2). A closer inspection of *Actinobacteria* and *Planctomycetes* azurin sequences strongly suggests these hits are due to contamination or wrong annotations (Supplementary Table S2); conversely, the few *Gemmatimonadetes* could represent a hint of a true presence of azurin in this clade, which is worthy of further investigation (Supplementary Table S2).

**Figure 1:**
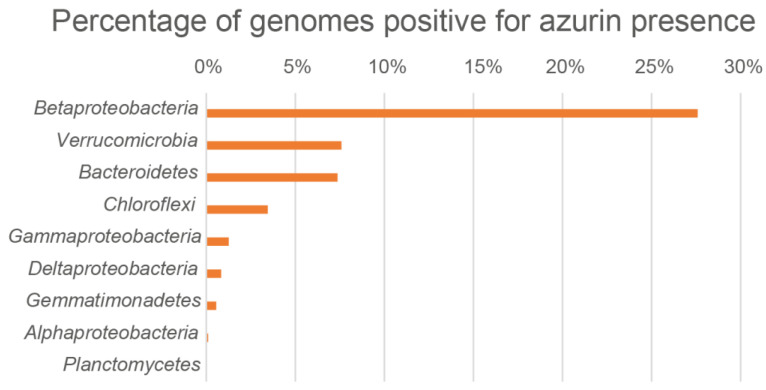
Percentage of genomes positive for azurin presence. The figure shows the percentage of genomes positive for azurin presence in each taxonomic group with at least one positive hit. Note that listed taxonomic groups have different rankings.

Even considering the bias in the taxon composition of the analyzed dataset, especially regarding the redundancy of *Proteobacteria* sequences, and the differences in screened genomes for the different phyla, *Betaproteobacteria* was the group with the highest presence of azurin genes, being 27.60% of the screened genomes positive for azurin presence (Table 1 and Figure 1). *Verrucomicrobia* and *Bacteroidetes* are, respectively, the second and the third phyla with similar percentage of azurin hits (more than 7%) (Figure 1).

**Figure 2:**
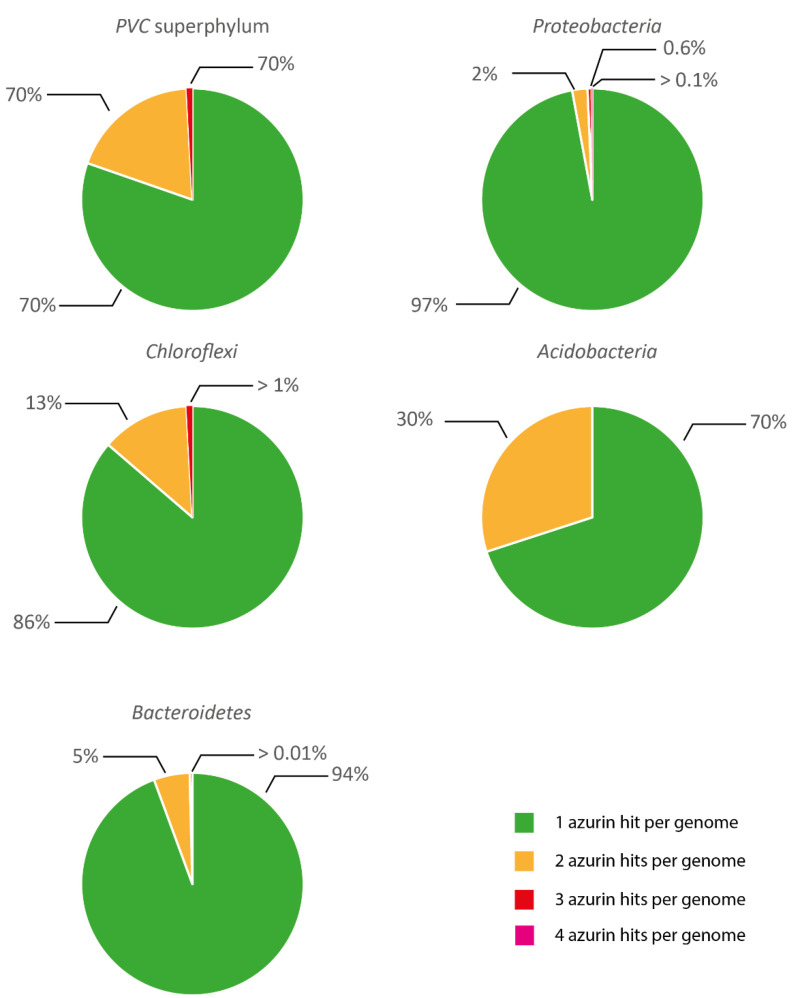
Number of azurin hits per genomes. Percentage of 1 azurin hit, 2 azurin hits, 3 azurin hits and 4 azurin hits on retrieved genomes.

Most of the analyzed genomes harbour a single copy of azurin-coding gene (Figure 2). Two or more paralogous copies per genome were detected in a few microorganisms and apparently were more frequent in representatives of *PVC* and *Chloroflexi*, even though this comparison should be taken cautiously due to the different number of screened genomes and the possible bias in taxon composition (especially for *Proteobacteria*).

Overall, data from Table 1 revealed an unexpected, scattered distribution of azurin across the domain Bacteria. We also mapped the presence of azurin in the phylogenetic tree reported by Hug et al. (2016) [36]. Data obtained are shown in Supplementary Figure S1, wherein the analysis confirmed the scattering of azurin on different and not-close branches of the tree. We also checked the presence of azurin in the genome of all representatives of a given taxon at different taxonomic level; we focused our attention on *Proteobacteria* (particularly on *Gamma*- and *Betaproteobacteria*). Data obtained concerning *Gammaproteobacteria* are shown in Table 2 and Supplementary Table S4, wherein the analysis revealed that within the same phylum, only some genera were positive for azurin presence. Similarly, within the same genus, neither all the species, nor all the representatives of each species, harbour the azurin-coding gene.

A quite different scenario appeared when the same analysis was performed on *Betaproteobacteria*, i.e., the group exhibiting the highest percentage of genomes harbouring the azurin-coding gene. Data reported in Supplementary Table S5 revealed that not all the betaproteobacterial genera were positive for azurin presence, similarly to gamma-proteobacteria. Conversely, and quite unexpected based on the gammaproteobacterial scenario, in each betaproteobacterial analyzed species, all the scanned genomes contained (at least) one copy of azurin coding gene.

To attempt to discern whether the scattering of azurin might be due either to its loss in some phyla or to horizontal gene transfer events, or both, a phylogenetic analysis of the azurin amino acid sequence was carried out (see below).

### 3.2. Phylogenetic Analysis of Azurin

The phylogenetic analysis was performed using all the retrieved sequences belonging to FCB group: PVC superphylum, *Acidobacteria*, *Chloroflexi*, *Actinobacteria*, *Alphaproteobacteria* and *Deltaproteobacteria*. Considering the extremely large numbers and taxonomic redundancy of sequences in *Gammaproteobacteria* and *Betaproteobacteria*, a selection of sequences was made in these groups so as to reduce the number of analyzed sequences, while maintaining an appropriate representativeness of the various sub-groups. A total of 2088 sequences of azurin-like genes were used, plus 62 sequences of auracyanin genes belonging to *Bacteroidetes* as outgroup (list of used sequences available in Supplementary Table S3).

**Figure 3:**
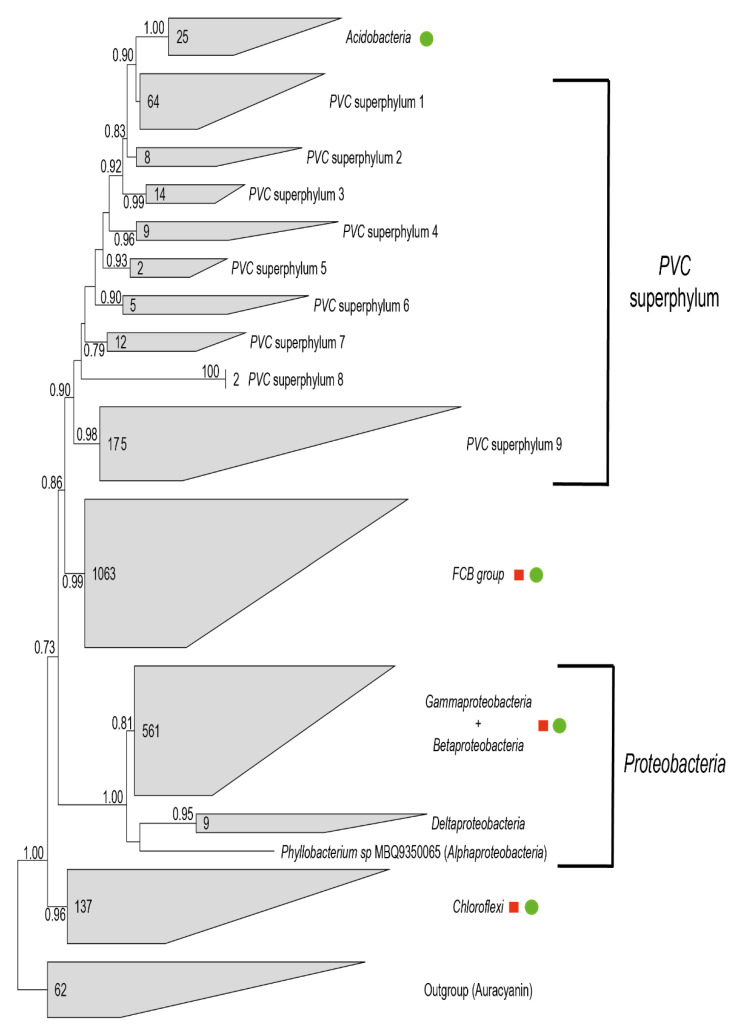
Phylogenetic analysis of retrieved azurin sequences. Maximum-Likelihood tree based on the amino-acidic sequences of the retrieved azurin sequences. Numbers on the nodes indicate the Shimodaira–Hasegawa (SH) test values for the relative nodes. Only values higher than 0.70 for the SH test were shown. Numbers inside the cluster indicate the number of sequences for each cluster. Red squares indicate the presence of *Alphaproteobacteria*-related sequences in the cluster, while green circles indicate presence of *Actinobacteria*-related sequences inside the cluster.

The phylogenetic analysis was able to retrieve a clear separation between the auracyanins used as an outgroup and the azurins (Figure 3).

The phylogenetic analysis was able to retrieve a clear separation between the auracyanins used as outgroup and the azurins (Figure 3). The Shimodaira–Hasegawa test values, reported on the tree, support the monophyly of the sequences belonging to FCB group, *Chloroflexi* and *Acidobacteria*. *PVC* members cluster all together, but the positioning of the group formed by the sequences belonging to *Acidobacteria*, prevent them from forming a single monophyletic group. All the sequences belonging to *Betaproteobacteria* and *Gammaproteobacteria* cluster together, even if not forming two separate monophyletic groups (data not shown). Most of the sequences belonging to the *Deltaproteobacteria* group cluster together, while *Alphaproteobacteria* are scattered among the other retrieved groups. *Actinobacteria*, as well, fail to form a monophyletic cluster.

Azurin sequences showed an overall mean identity of 38% (Table 3). As expected, there is a higher level of identity inside the various groups.

### 3.3. Azurin Operon Prediction in Selected Organisms

The analysis made with the operon prediction software on the random selection of complete genomes (10 Bacteroidetes genomes, 10 Proteobacteria genomes, 5 PVC genomes, 2 Acidobacteria genomes) depicted a situation in which the azurin gene generally did not belong to operon structures (Supplementary Table S4). Indeed, in the available PVC complete genomes, only in 1 case out of 8 was azurin found in a multigene operon, and similarly, in Acidobacteria (no case of multigene operons) and selected Proteobacteria (1 case out of 10). On the other hand, in selected Bacteroidetes, the number of multigene operons was apparently much higher (6 cases out of 10). In very few cases, azurin genes were found in multigene operons composed by more than 2 genes (Supplementary Table S3). With the analyzed selection, we could not observe a clear pattern of organization among the various phylum-like clusters, and azurin-associated genes were always different from each other, with the single exception of the azurin operons found in 2 members of family Flavobacteriaceae (Bacteroidetes, Flavobacteria) (ACC CP033068 and ACC CP0718), in which azurin gene was found in a 2 gene operon associated with the same predicted transcriptional regulator (Supplementary Table S4). Notably, in the case of Opitutus terrae strain PB90-1 (ACC CP001032), 2 different azurin genes were present. One was organized in a single gene operon, while the other in a 2 gene operon.

### 3.4. Azurin Conserved Domains Search and Analyzes

**Figure 4:**
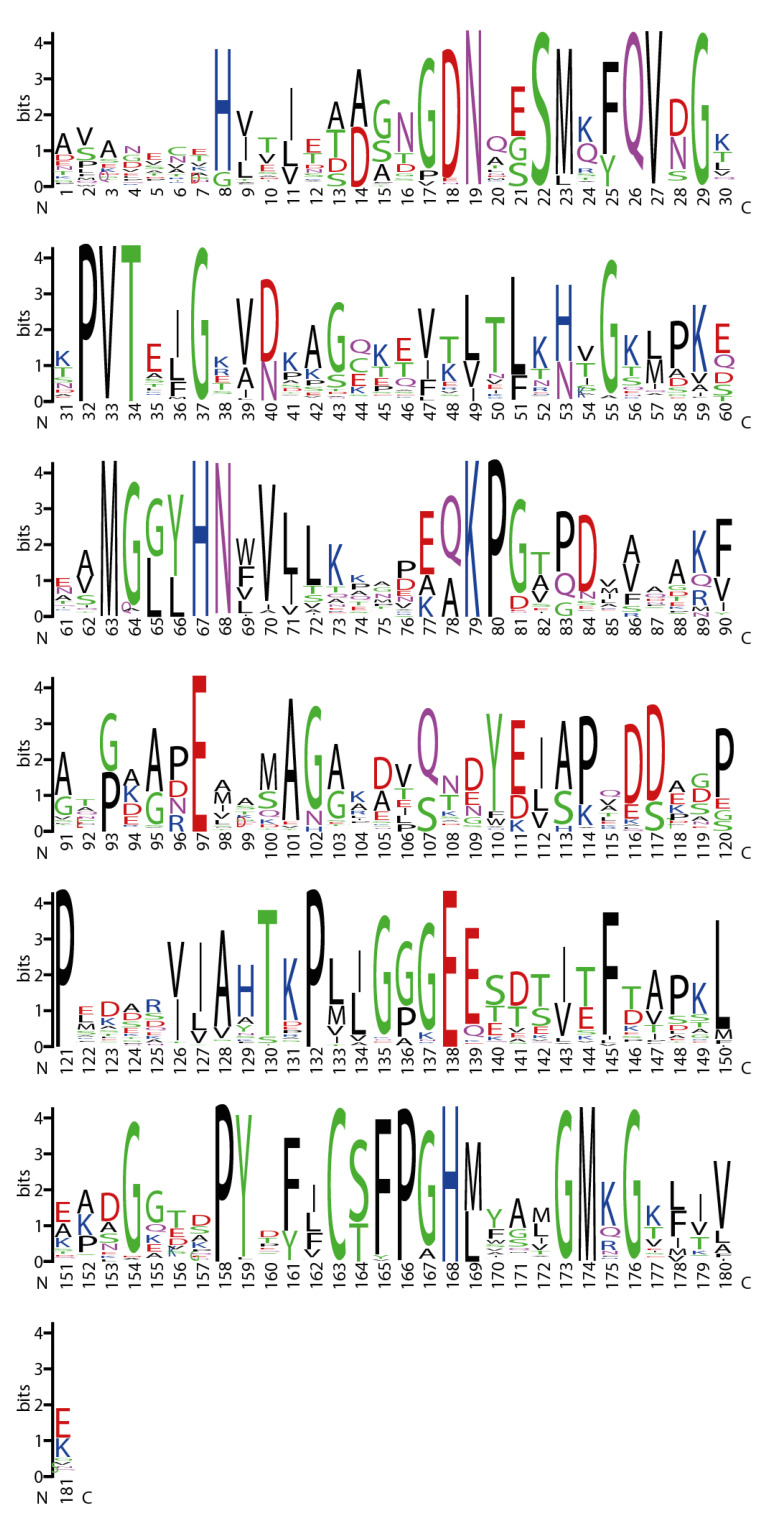
Logogram of the longest conserved domain shared by all the azurin-like sequences. Size of each letter corresponds to the frequency of the related amino acid in that position. Numbers on the X axis refers to the position of the retrieved alignment.

The analysis of the multi-alignment of (all) the azurin amino-acid sequences showed a greater divergence in the N-terminal region, with no clear conserved domain shared by all the analyzed sequences, while it was possible to identify a larger region (about 130 amino acids), which is rather conserved among all the investigated sequences at the C-terminus (Figure 4).

**Figure 5:**
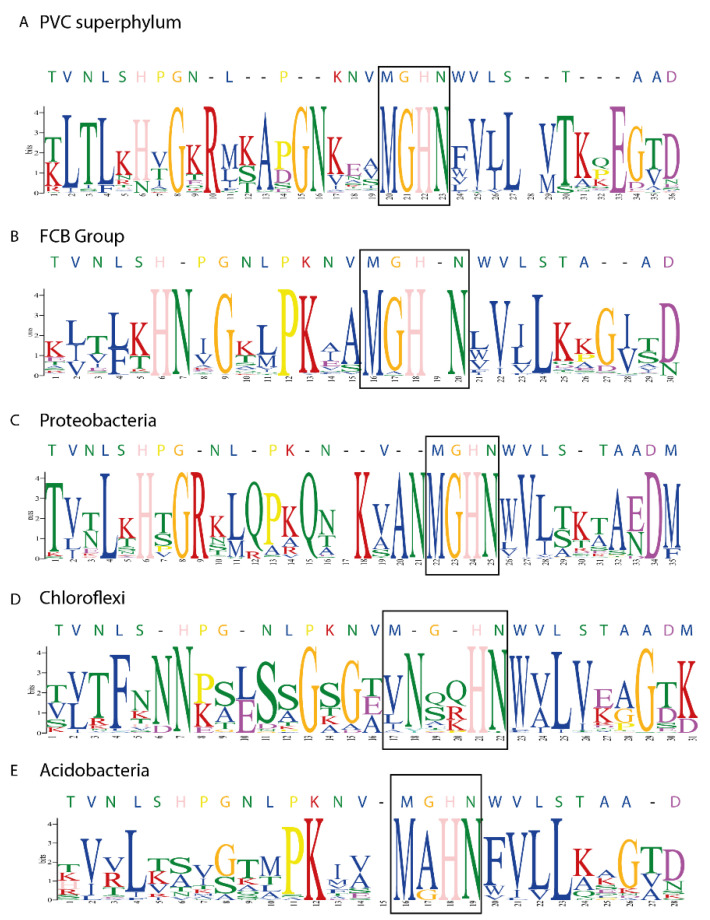
Logogram of p28 domain found in sequences of the major clusters. Logogram of the retrieved p28 domain in PVC superphylum (**A**), FCB group (**B**), Proteobacteria (**C**), Chloroflexi (**D**) and Acidobacteria (**E**). Size of each letter corresponds to the frequency of the related amino-acid in that position. Numbers on the X axis do not refer to the position on the sequence, but to the position of the retrieved alignment. Letters on top of each figure represent the corresponding amino-acid in the P. aeruginosa p28 domain. Black square frame indicates the MGHN tetrapeptide.

Due to the importance played by the P. aeruginosa p28 fragment in the mechanism related to the selective entrance of this protein inside cancer cells, we also focused our attention on its presence and conservation degree among the various groups of organisms. The data obtained revealed that in all the analyzed groups it was possible to detect the presence of the p28 fragment (Figure 5). The domain showed, overall, a good degree of conservation among the various phyla, with several amino acids that are highly conserved. In particular, the central tetrapeptide MGHN was almost perfectly conserved in Protoebacteria, FCB Group, PVC superphylum and, to a lesser extent, in Acidobacteria.

Nevertheless, just 8 of the retrieved sequences from the phylum Chloroflexi were positive for its presence. Moreover, the p28 domains retrieved from the phylum Chloroflexi were more divergent in respect to the domains present in the other phyla, with even the MGHN tetrapeptide less conserved (Figure 5D).

## 4. Discussion

The quest for new and most efficacious tools in the long-lasting fight against cancer diseases, involves the study of microbial molecules. During the last two decades, these kinds of molecules had proven to be a great source of possible, new candidates for anticancer molecules, and several of them are currently applied in anticancer therapies [3,4,5]. The azurin produced by P. aeruginosa is a well-known copper-binding protein, whose activity as an anticancer agent, as well as that of the related p28 fragment, have been assessed in the last two decades [17,21,37,38]. Despite P. aeruginosa azurin being extensively investigated from a functional and structural viewpoint, very little is known about its evolutionary history. This work represents the first report in which the screening for azurin genes was investigated not only in P. aeruginosa, but also for considering all the available genomes published up to now. Data obtained in this work revealed that neither Archaeal nor Eukaryotic azurin sequences were retrieved, strongly suggesting that the encoding gene was not part of the genome of the Last Universal Common Ancestor and that its appearance might be traced somewhere in the domain Bacteria. The phylogenetic distribution of azurin revealed for the first time that the azurin gene was found not only within the Pseudomonas genus, but also within almost the whole Proteobacteria phylum and, additionally, in representatives of several other major phyla (Table 1). The azurin gene is present in some but not all bacterial taxa; as an example, it is completely absent in Epsilonproteobacteria, Cyanobacteria, Firmicutes, Tenericutes and Chlamidiae, even though a very high number of genomes were scanned (see Table 1). In addition to this, the azurin-coding gene is not present in all the gamma-proteobacterial species of the same genus nor in all representatives of the same species, whereas, all the representatives of the beta-proteobacterial species positive for azurin presence, harbour the encoding gene.

Accordingly, at least two different scenarios can be envisaged to explain this scattering: (i) the first one predicts that the azurin-coding gene appeared in the last common ancestor of Bacteria and was lost in some phylogenetic phyla and in different species of the same genus and/or in different members of the same species; (ii) the alternative scenario predicts that it appeared in one branch and was then horizontally transferred between microorganisms belonging to different taxa. It is worth mentioning that the two scenarios are not mutually exclusive, i.e., a mixed scenario could be envisaged with mainly vertical inheritance and gene loss with some cases of horizontal gene transfer.

To discern between these alternatives, a phylogenetic analysis performed using a subset of the azurin amino-acid sequences was carried out, which failed to properly reconstruct high level taxonomic relationships (i.e., family level relationships or genus level relationships) among the investigated organisms, mainly due to the lack of a sufficiently robust phylogenetic signal. Nevertheless, sequences derived from the same taxa generally clustered together, suggesting that vertical inheritance apparently was the major force in shaping the evolutionary history of those genes. However, it is possible that recent Horizontal Gene Transfer (HGT) events between far-related groups occurred (Figure 3), as suggested by the presence of Alphaproteobacterial and/or Acidobacterial azurin sequences in different branches of the azurin tree and/or by the intermixing of Beta- and Gammaproteobacterial sequences.

Accordingly, the absence of a shared organization (i.e., the absence of a shared operon and the difference in the surrounding genomic landscape) can also point to the occurrence of parallel HGT phenomena that contribute to increase the diversification and the wide spreading among the various organisms. This observation might represent one of the possible explanations for the positioning of the sequences belonging to Alphaproteobacteria and Actinobacteria. However, we cannot a priori exclude the possibility that other possible explanations might be taken in account to explain the positioning of those sequences, such as possible taxonomic mis-annotation of the related genomes [39], or azurin presence as a result of contamination from different organisms during the genome reconstruction, given the incomplete assembling status of the retrieved organisms [40], such as in the case of Actinobacteria and several Alphaproteobacteria contigs that we analyzed in more detail (see Table 2). Lastly, the positioning of the few sequences related to Alphaproteobacteria and Actinobacteria might also be due to this extremely low taxon sampling, which may affect the phylogenetic reconstruction [41].

Furthermore, a similar conclusion can be considered to interpret the positioning of azurin retrieved from the Acidobacteria group. In this case, either a HGT phenomenon from a representative of phylum Verrucomicrobia to a representative of phylum Acidobacteria, or an artifactual reconstruction due to a phenomenon of Long Branch Attraction [42], which wrongly positions the whole Acidobacteria cluster of sequences, may be at the base of the strange positioning of these sequences.

On the basis of the available data, it is still not possible to explain why this gene exhibits so scattered a distribution. A preliminary inspection of Bacteria harbouring the azurin gene revealed that, in most cases, these bacteria interact with eukaryotic organisms (both plants and animals). However, this will require a deeper understanding of the ecological role played by azurin, an issue that is beyond the scope of this manuscript.

Concerning the origin of the azurin-coding gene, according to Kesse and Gibbs (1992) [43], new genes can be generated at least in two different ways: (i) polynucleotide molecules can be synthesized de novo or (ii) or from pre-existing molecules, through a mechanism referred to as “overprinting”. Moreover, at least another mechanism (in addition to the well-known gene duplication followed by evolutionary divergence) can be invoked to explain the origin of extant genes from simpler ones; this molecular mechanism is referred to as gene elongation, i.e., in tandem duplication of a gene followed by the deletion of the intervening sequence and the transformation of the nonsense codon of the first gene into a sense codon, resulting in an elongated gene double the size of the ancestor gene. Concerning the latter mechanism, the analysis of the amino-acid sequence of azurin proteins from different bacteria (not shown) did not reveal the existence of internal regions sharing a degree of sequence-similarity sufficiently high to suggest that the encoding gene might be the outcome of one (or more) gene elongation event(s) [44,45]. It is also known that azurin belongs to the Cupredoxin protein family, which, in turn, includes auracyanins, amicyanins, azurins, rusticyanins, pseudoazurins, and halocyanins. The analysis of these proteins did not reveal an extensive degree of sequence similarity among them along their entire sequence (see Introduction); this might suggest that if they belong to a paralogous gene family, the duplication event(s) leading to the present-day genes should have occurred in the early stages of bacterial evolution.

The shared presence of the p28 domain among the various clades, although with different consensus sequences (Figure 5), is also interesting. The overall similarity and the maintenance of some key amino acids seems to be a strong hint of a similar role played also in other groups. Representatives of phylum Chloroflexi seems to be, at least partially, an exception to this finding, since the p28 domain is absent in several of the azurin proteins. However, this is coherent with the high degree of divergence of azurin-like protein of this group in respect to the others, and with the early branching positioning of the Chloroflexi clade in our phylogenetic analysis. It would be of great interest to investigate whether the p28 domains found in other taxonomic groups are able to show any anticancer activity such as the one in P. aeruginosa, as the overall similarity in consensus sequences seems to suggest.

Indeed, the finding of azurin proteins in such a heterogeneous array of organisms can open new perspectives in this research field. As an example, Akkermansia muciniphyla, a member of the phylum Verrucomicrobia belonging to the family Akkermansiaceae is an inhabitant of the intestinal epithelial crypts also in human gut [46]; this bacterium is known to play a key role in maintaining gut health, and its population dynamics has been related to the occurrence of several diseases, including obesity [47] and inflammatory bowel disease [48]. Moreover, Akkermansia muciniphyla has also gained great attention in the medical field during recent years, as a probiotic during colon and gut anticancer therapy [49]. Another intensively studied phylum in the human gut microbiome is that of Bacteroidetes. Bacteroidetes are known to play a fundamental role in regulating gut functioning and health, and a reduced abundance of members of this phylum has been associated to several weight disorders, including obesity [50] and diabetes [51]. Moreover, in recent years, a correlation between Bacteroidetes abundance in the gut microbiota of breast cancer-affected patients [52] and microbiota of lung cancer-affected patients [53] has been demonstrated, underlying the important role played by these organisms in such kinds of cancer diseases.

The fact that our screening was able to identify the azurin protein in members of the same Verrucomicrobia family, as A. muciniphyla, as well as in many representatives of Bacteroidetes, may also represent a first step for further analyzes in the direction of depicting the mechanisms underlying the role played by these organisms in relation to cancer diseases, and also reveal new, possible tools to be employed in medical and research fields. The need to evaluate possible biological activities (e.g., anticancer) of p28 from Verrucomicrobia or Bacteroidetes is of special interest if we consider that representatives from these groups are already known and well-studied components of human gut microbiome and involved in prognosis of cancer treatment.

Hence, in this work, other than deciphering the taxonomic distribution of the azurin gene in bacteria, we opened the way for further investigations aimed at testing the biological activity of azurin/p28 domain from other bacterial taxa other that P. aeruginosa. In light of the already obtained results for azurin/p28 of P. aeruginosa against many types of cancer, this perspective acquires even more importance and interest in considering different kinds of tumors, especially those for which commonly used drugs have not, up to now, exhibited a satisfactory effect in terms of regression and survival.

## Figures and Tables

**Table 1:** Screening results for the presence of azurin in the available bacterial genomes. (A) Phyla/Superphyla positive for azurin presence. List of phyla or Superphyla found positive for the presence of azurin. For each phylum the number of analyzed genomes is reported together with the number (n) of positive hits for azurin. (B–D) Number of hits found in the various phyla. Number of hits for phylum Proteobacteria (B), FCB group (C), PVC superphylum (D) and Terrabacteria group (E). Numbers of genomes for each class/phylum is reported as well as number of hits.

	Phylum/Superphylum	n of Genomes	n of Hits	n of Positive Genomes	% of Positive Genomes
**A**	*Proteobacteria*	693,126	13,546	12,942	1.86
	*Acidobacteria*	887	26	20	2.25
	FCB group	15,401	1111	1038	6.73
	*PVC* superphylum	7365	288	239	3.24
	*Terrabacteria* group	173,867	140	123	0.07
	**Class**	**Proteobacteria**			
**B**	*Betaproteobacteria*	19,552	5857	5397	27.60
	*Gammaproteobacteria*	595,179	7689	7544	1.26
	*Deltaproteobacteria*	2271	19	19	0.83
	*Alphaproteobacteria*	12,724	14	14	0.11
	*Epsilonproteobacteria*	63,366	0	0	0
	Others	32	0	0	0
	**Phylum**	**FCB group**			
**C**	*Bacteroidetes*	14,020	1063	1034	7.37
	*Gemmatimonadetes*	723	4	4	0.55
	Others	658	0	0	0
	**Phylum**	***PVC*** **superphylum**			
**D**	*Verrucomicrobia*	3147	287	238	7.59
	*Planctomycetes*	2200	1	1	<0.01
	*Chlamydiae*	2009	0	0	0
	**Phylum**	***Terrabacteria*** **group**			
**E**	*Chloroflexi*	3388	134	117	3.45
	*Actinobacteria*	21,609	6	6	<0.01
	*Cyanobacteria*	1794	0	0	0
	*Firmicutes*	145,162	0	0	0
	*Tenericutes*	1173	0	0	0
	Others	742	0	0	0

**Table 2:** List of bacterial genera belonging to the *Gammaproteobacteria* harboring the azurin-coding gene. Number of hit in each genus is reported. The “Bioreactor sample” entry has been included in the table even if it was not a genus, just for a matter of completeness.

Genus	n of Hits	Genus	n of Hits
*Pseudomonas*	4313	*Paraglaciecola*	1
*Dyella*	2	*Steroidobacter*	2
*Dokdonella*	1	*Xylella*	89
*Rhodanobacter*	1	*Imhoffiella*	3
*Ventosimonas*	1	*Methylophaga*	2
*Halomonas*	9	*Pseudoxanthomonas*	9
*Alteromonas*	18	*Salinivibrio*	5
*Oblitimonas*	3	*Alcanivorax*	2
*Pseudomonadaceae*	4	*Nitrincola*	2
*Lysobacter*	9	*Kangiella*	3
*Cellvibrio*	2	*Salinisphaera*	1
*Pseudoalteromonas*	15	*Fulvimonas*	1
*Aeromonas*	304	*Pseudoteredinibacter*	2
*Oceanimonas*	2	*Arenimonas*	1
*Oceanisphaera*	1	*Pseudofulvimonas*	1
*Shewanella*	81	*Pseudidiomarina*	1
*Vibrio*	1432	*Glaciecola*	1
*Xanthomonas*	566	*Vulcaniibacterium*	1
*Idiomarina*	6	*Dichelobacter*	1
*Stenotrophomonas*	267	*Bioreactor sample*	1
*Luteimonas*	5		

**Table 3:** Mean identity among azurin sequences. Identity referred to sequences for each of the analyzed phylum/supergroup and among all the sequences.

Phylum/Superphylum	Mean Identity
*Acidobacteria*	48%
*Chloroflexi*	41%
FCB group	45%
*Proteobacteria*	62%
*PVC* superphylym	44%
ALL	38%

## Data Availability

The data presented in this study are openly available in NCBI database (https://www.ncbi.nlm.nih.gov/, accessed on 15 November 2021).

## Supplementary Materials

The following are available online at https://www.mdpi.com/article/10.3390/microorganisms10010009/s1, Figure S1: Distribution of Azurin genes among the main bacterial lineages; Table S1: List of retrieved hits for Azurin gene, Table S2: Retrieved azurin sequences checked for taxonomic annotation, Table S3: Selected Azurin sequences used for the phylogenetic analyzes, Table S4: List of genomes screened for presence of azurin operon, Table S5: Number and percentage of genomes of Gammaproteobacteria harboring the azurin coding gene, Table S6: Number and percentage of genomes of Betaproteobacteria harboring the azurin coding gene belonging to different species.
