# Supplementary material for: The Azurin Coding Gene: Origin and Phylogenetic Distribution
Source: Microorganisms. 2021 Dec 22;10(1):9. doi: 10.3390/microorganisms10010009 (PMC8779525; doi:10.3390/microorganisms10010009)
Supplement: Supplementary file 1 [file microorganisms-10-00009-s001.zip › Supplementary Figure S1.pdf]

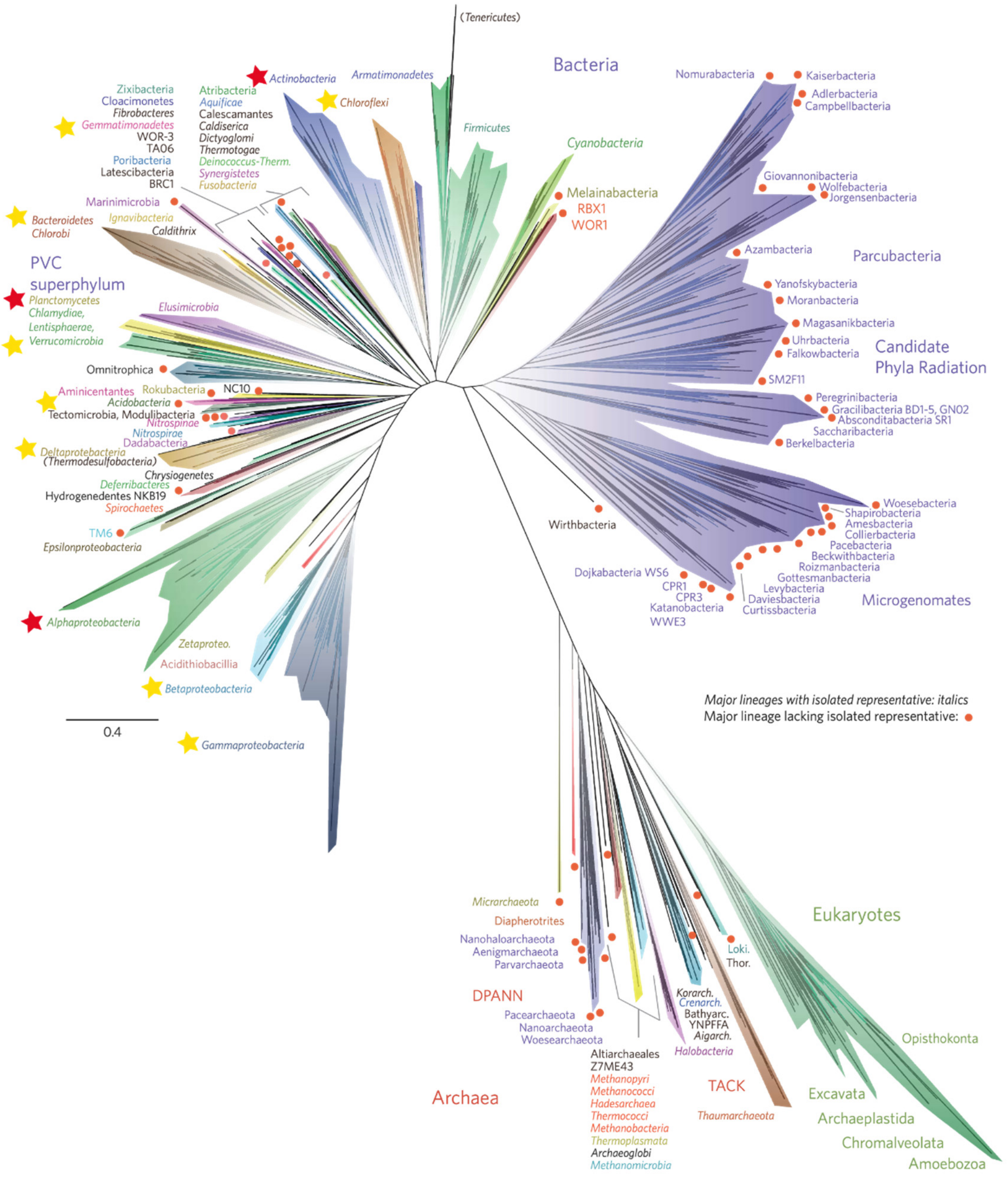

Figure S1. Distribution of Azurin genes among the main bacterial lineages. Yellow stars indicate lineages in which azurin genes have been found, while red star indicate lineage in which Azurin had been its presence may be due to assembly contamination or misannotation. Modified from Hou et al. 2016.
